# Genosomes: An Introspection into Transfection, Future Perspectives and Applications

**DOI:** 10.34172/apb.025.43191

**Published:** 2025-09-09

**Authors:** Abhirami Subramony, Divyatha Raj, Fiza Fairooz, Aparna Venugopal, Aparna Rajesh, Sreeja C Nair

**Affiliations:** Amrita School of Pharmacy, Amrita Institute of Medical Sciences, Amrita Vishwa Vidyapeetham, Kochi-682041, India

**Keywords:** Genosomes, Lipoplexes, Cationic liposome, Transfection efficiency

## Abstract

The advent of advanced gene delivery platforms has transformed the precision targeting of therapeutic nucleic acids, such as miRNA, siRNA, and pDNA, for the treatment of genetic and acquired diseases, including cystic fibrosis, malignancies, sickle cell anaemia, and β-thalassemia. Although viral vectors have traditionally dominated this field, non-viral systems, particularly genosomes (cationic lipid-based nanocarriers or lipoplexes), have emerged as promising alternatives due to their enhanced biosafety, lower immunogenic potential, and manufacturability. These nanostructured systems facilitate efficient nucleic acid condensation, protect against enzymatic degradation, and enhance cellular uptake and endosomal escape. Further refinements, including PEGylation, incorporation of helper lipids, and stimuli-responsive formulations, have significantly improved transfection efficiency and tissue-specific delivery. Notable clinical advancements, such as RNA-lipoplexes in cancer immunotherapy and multifunctional envelope-type nanodevices (MEND), highlight their therapeutic potential. This review provides a critical analysis of genosome design strategies, formulation techniques, intracellular trafficking mechanisms, clinical applications, patented innovations, and future prospects to advance genosome-mediated gene therapy.

## Introduction

 Nucleic acid-based therapeutics have emerged as a transformative modality in modern medicine, offering novel interventions for both inherited and acquired diseases.^1,2^ These therapies utilise functional genetic materials, such as plasmid DNA (pDNA), small interfering RNA (siRNA), and microRNA (miRNA), to modulate pathological gene expression by restoring defective genes, silencing aberrant transcripts, or reprogramming dysregulated cellular pathways.^3^ Despite their therapeutic potential, the clinical translation of nucleic acid drugs faces significant delivery challenges.^4,5^ Unmodified nucleic acids exhibit poor pharmacokinetic profiles due to their high molecular weight, anionic charge, and hydrophilicity, which hinder cellular uptake.^6^ Additionally, susceptibility to enzymatic degradation and electrostatic repulsion by negatively charged cell membranes further diminishes bioavailability and therapeutic efficacy. While localised administration, such as intramuscular or intratumoral injection, can enhance tissue-specific gene expression, these methods lack the systemic applicability required for many disorders. Consequently, engineered gene delivery vectors have become essential to protect nucleic acids from degradation, enhance cellular internalisation, and enable targeted tissue delivery.

 Among the various delivery platforms, lipid-based vesicular systems have gained prominence due to their capacity to encapsulate genetic payloads, improve biodistribution, and facilitate controlled release. Liposomes, nanoscale spherical vesicles composed of lipid bilayers surrounding an aqueous core, represent one of the most extensively studied lipid-based carriers. Their amphiphilic nature allows efficient encapsulation of both hydrophilic and hydrophobic therapeutics, making them versatile vehicles for nucleic acid delivery. Early approaches incorporated viral vectors, including retroviruses and adenoviruses, into liposomal formulations to leverage their high transfection efficiency and nuclear localisation capabilities.^7,8^ However, despite their initial clinical promise, viral vectors are limited by immunogenicity, insertional mutagenesis (a potential oncogenic risk), toxicity and the challenges associated with large-scale production.^9^ These limitations have spurred the development of non-viral alternatives, including cationic lipids, polyethyleneimine (PEI)-based polymers, dendrimers, biodegradable polymeric nanoparticles such as PLGA, and cell-penetrating peptides, which offer improved safety, scalability, and tunable physicochemical properties.^10^

 A leading non-viral strategy involves the use of genosomes (or lipoplexes), which are formed through electrostatic complexation between cationic lipids and anionic nucleic acids. These systems present several advantages, including minimal immunogenicity, cost-effective manufacturing, and the ability to preserve the structural and functional integrity of genetic cargo during delivery. Genosomes have demonstrated therapeutic efficacy in treating monogenic disorders (e.g., sickle cell anaemia, β-thalassemia, and cystic fibrosis) as well as various malignancies, including non-small cell lung cancer and breast cancer.^11^ Current research focuses on optimising genosome formulations through surface modifications, including pegylation and ligand conjugation, and co-delivery with endosomolytic agents to enhance transfection efficiency and tissue specificity.^12^ With continuous advancements, genosomes represent a clinically viable and versatile platform for next-generation gene therapy, bridging the gap between preclinical innovation and therapeutic application.

## Viral vectors v/s non-viral vectors

 Viral vectors exhibit unparalleled transfection efficiency, yet their clinical application is hampered by critical safety limitations, including immunogenic responses, cytotoxic effects, and the potential for insertional mutagenesis, a process wherein viral genome integration disrupts tumour suppressor genes or activates proto-oncogenes, increasing the risk of malignant transformation.^13,14^ In contrast, non-viral delivery systems offer a safer and more controllable alternative, characterised by low immunogenicity, absence of pathogenic risks, cost-efficient manufacturing, and improved biosafety profiles. These advantages, combined with their scalability and long-term stability, have catalysed a shift toward non-viral gene delivery platforms since the early 21^st^ century. A comparative analysis of viral and non-viral vectors is presented in Table 1. While viral vectors, such as adeno-associated viral (AAV), adenovirus (AdV), lentivirus (LV), bacteriophage, and herpes simplex virus (HSV), remain widely utilised in research, non-viral systems are gaining traction in clinical settings. These include lipid-based carriers such as liposomes, cationic polymers (e.g. PEI), inorganic nanoparticles (e.g. gold and silica nanostructures), and endogenous nanovehicles like exosomes and RBC membrane-derived vesicles. Their emerging therapeutic applications are systematically outlined in Tables 2 and 3.

**Table 1:** Highlighted differences between viral and non-viral vectors

**Viral vectors**	**Non-viral vectors**
Transfection efficiency is high	Transfection efficiency is low
Causes immunogenicity and cytotoxicity	It does not cause immunogenicity and cytotoxicity
Decreased bio-safety	Increased bio-safety
High cost and difficult production	Low cost and ease of production
Use has gradually decreased due to toxicity	Use has increased in recent years
Not safe to store	Safe to store

**Table 2:** The most commonly used viral vectors

**Vectors**	**Family**	**Advantages**	**Disadvantages**	**Ref**
Adenoviral	Adenoviridae	Titers are large. Can move the transgene into dividing and normal cells	Transgene expression occurs in a short period and is immunogenic.	^15^
AAV	Parvoviridae	Abundant host availability, sustainability	It can produce toxicity, and its packaging capability is restricted.	^16^
Retroviral	Retroviridae	Safe, low immunogenicity	Titers are low. Useful for actively dividing cells only. Chances of insertional mutagenesis	^17^

**Table 3:** The most commonly used non-viral vectors

**Non-viral vectors**	**Example **	**Gene payloads**	**Advantages **	**Applications**	**Ref **
Lipid nanoparticles	Ionisable LNPs (Dlin-MC3-DMA)	mRNA, siRNA, miRNA, pDNA	High encapsulation efficiency Higher expression of nucleic acids	Vaccines (e.g. COVID-19), siRNA therapeutics (e.g. Patisiran)	^18^
Polymeric nanoparticles	PEG-PEI, PLGA	pDNA, CRISPR-Cas9, shRNA	High transfection *in vitro * Lower cytotoxicity	Cancer gene therapy, regenerative medicine	^19^
Inorganic nanoparticles	Gold nanoparticles, mesoporous silica	siRNA, ASO, mRNA	High cellular uptake Higher nuclease resistance	Targeted therapy like tumour-specific delivery	^20^
Cell-penetrating peptides	TAT peptide, Penetratin	CRISPR RNP, siRNA, pDNA	Higher nuclear localisation High endosomal escape rate	Neurological disorders, genome editing	^21^
Hydrogels/ Hybrid systems	Chitosan-hyaluronic acid	mRNA, pDNA, miRNA	Sustained release High retention at the injection site	Tissue engineering, localised therapy	^22^

## Nucleic acids utilised in Genosome-based delivery systems

 The selection and optimisation of nucleic acid payloads represent a critical determinant in the efficacy of genosome-based gene delivery systems, with each class of therapeutic oligonucleotides, including siRNA, miRNA, ASOs, and CRISPR-Cas9 components, presenting unique physicochemical properties and delivery challenges that necessitate tailored formulation strategies (Table 4).

**Table 4:** Comparative analysis of nucleic acids for delivery by genosomes

**Category **	**siRNA**	**miRNA**	**ASOs**	**CRISPR-Cas9 system**
Size (nt/kb)	20-25	20-22	15-25	gRNA: ~100 Cas9: ~4000
Charge	Strongly negative	Negative	Modifiable	Negative (RNA) Variable (Protein)
Key delivery challenge	Nuclease sensitivity Endosomal escape	Tissue specificity Off-target effects	Nuclear delivery Biodistribution	Payload size Immunogenicity
Genosome as a delivery vehicle	Ionisable LNPs Cationic complexation	Targeted formulations PEGylation	Stabilised formulations Nuclear localisation signals	Co-delivery systems pH-sensitive lipids
Clinical relevance	FDA-approved formulations, e.g. Patisiran	MRX34	FDA-approved formulations, e.g. Nusinersen	NTLA-2001
Therapeutic advantage	Potent silencing Reversible effect	Multi-gene modulation Natural regulator	Splice modulation Chemical versatility	Permanent editing Precision targeting
Optimal lipid composition	Dlin-MC3-DMA DSPC: Chol: PEG	DOTAP: DOPE Targeted PEG-lipids	Neutral/stealth lipids Phosphorothioate analogs	Cationic: neutral blends Helper lipids
Reference	^23^	^24^	^25^	^26^

###  Small interfering RNA (siRNA)

 siRNA has become a prime candidate for genosome-mediated delivery owing to its well-defined RNA interference (RNAi) mechanism and compact molecular structure. The 20-25 base pair duplexes readily form stable complexes with cationic lipids via electrostatic interactions, enabling efficient encapsulation within lipid nanoparticles (LNPs).^27^ Genosomes overcome major siRNA delivery barriers by providing nuclease protection during systemic circulation and enhancing cellular uptake through charge-mediated endocytosis.^28,29^ Advanced formulations employ ionisable lipids (e.g. Dlin-MC3-DMA) that undergo pH-dependent protonation in endosomes, facilitating membrane destabilisation and cytosolic siRNA release. This strategy has achieved clinical validation with Patisiran (Onpattro^®^), an FDA-approved genosome, delivered siRNA therapeutic for transthyretin-mediated amyloidosis that demonstrates effective hepatocyte-specific gene silencing.^30^

###  MicroRNA (miRNA)

 As endogenous post-transcriptional regulators, miRNA presents both therapeutic opportunities and delivery challenges for genosome systems.^31^ While their ability to modulate multiple disease pathways simultaneously is advantageous, the risk of off-target effects necessitates precise tissue targeting.^32^ Contemporary genosome designs address these requirements through surface-conjugated targeting ligands (e.g. folate, RGD peptides) for cell-specific delivery, and PEGylation to enhance circulatory half-life by minimising reticuloendothelial clearance. The amphiphilic nature of genosomes permits stable incorporation of miRNA mimics or antagomirs while preserving biological activity.^33^ Current optimisation efforts focus on lipid composition refinements to improve tissue accumulation profiles, as exemplified by clinical-stage candidates like MRX34 for oncology applications.

###  Antisense oligonucleotide (ASO)

 Genosome encapsulation significantly enhances the therapeutic potential of ASOs by addressing their delivery limitations.^34^ Although chemical modifications like phosphorothioate backbones, 2’-O-methyl groups improve ASO stability and target affinity, they remain insufficient for efficient intracellular delivery.^35^ Genosome formulations provide comprehensive solutions by protecting ASOs from serum nucleases, promoting cellular internalisation through optimised surface charge, and facilitating nuclear localisation via incorporated targeting motifs.^36,37^ While ASO therapies like Nusinersen (Spiranza^®^) for spinal muscular atrophy have demonstrated clinical success, genosome delivery could further enhance tissue distribution and pharmacokinetics, potentially reducing dosing frequency.^38^

###  CRISPR/Cas9

 The CRISPR/Cas9 platform presents unique delivery challenges that genosomes are particularly equipped to address.^39^ Unlike smaller nucleic acids, CRISPR components require co-delivery of both guide RNA (~100 nt) and Cas9 protein/mRNA (~4 kb coding sequence), demanding substantial payload capacity. Genosomes meet these requirements through cationic lipid-mediated nucleic acid condensation, ionisable lipid-facilitated endosomal escape, and optimised lipid ratios for particle stability.^40^ While clinical trials have demonstrated successful hepatic delivery, achieving efficient extrahepatic targeting remains challenging.^41^ Current research focuses on improving tissue specificity and reducing immune recognition.

## Structure of a genosome

**Figure 1:**
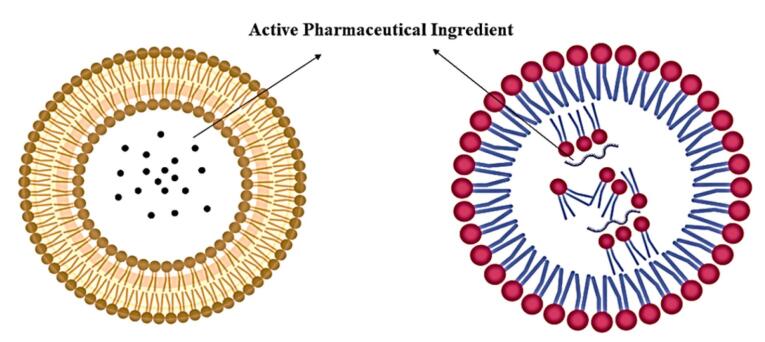
Structures of liposome and lipid nanoparticle

 Genosomes represent a class of synthetic, lipid-based nanocomplexes engineered for nucleic acid delivery, comprising three essential components- a cationic lipid, a neutral helper lipid, and the therapeutic payload (DNA or RNA).^42,43^ These nanostructures derive their stability from electrostatic interactions between the positively charged lipid moieties and the anionic phosphate backbone of the nucleic acids, thereby ensuring both structural integrity and protection of the genetic cargo.^44,45^ The cationic lipoplex system, a fundamental architectural element of genosomes, enhances transfection efficiency through charge-mediated interactions.^46^ The positively charged nanoparticle surface facilitates cellular uptake via association with negatively charged cell surface proteoglycans. Following internalisation, controlled disassembly of the complex enables targeted release of the nucleic acid payload, either to the cytoplasm (for RNA-based therapeutics) or the nucleus (for DNA-based therapeutics), to mediate gene silencing or expression. At the molecular level, genosomes adopt a spherical bilayer morphology, wherein amphiphilic lipids self-assemble with their hydrophobic tails oriented outward and hydrophilic headgroups inward (Figure 1). The cationic character of these lipids, typically conferred by primary or quaternary ammonium groups, serves dual functions- electrostatic condensation of nucleic acids, and promotion of cellular uptake.^47,48^ Notably, the number and chemical nature of these ammonium groups critically influence both nucleic acid compaction efficiency and overall transfection performance.

 Genosomes exhibit dynamic phase behaviours, predominantly adopting lamellar (Lα) or inverted hexagonal (H_II) arrangements at the nanoscale (10-50 nm), irrespective of the preparation method. In the lamellar phase, nucleic acids intercalated between parallel lipid bilayers in a rod-like conformation, with membrane fluidity modulated by temperature and lipid composition.^49^ H_II phase organises nucleic acids within a two-dimensional hexagonal lattice, while the micellar hexagonal phase localises them in interstitial, honeycomb-like regions.^50,51^ More complex architectures, including cubic and bicontinous phases, have also been documented.

 At larger length scales (100 nm to 1 μm), genosome morphology is dictated by preparation parameters such as mixing order and assembly conditions.^52,53^ While nanoscale organisation governs transfection efficiency, macroscale features influence critical *in vivo *performance metrics, including circulation half-life, biodistribution, and plasma stability.^54,55^

 The structural organisation of genosomes is primarily described by two theoretical models.^56,57^ The external binding model proposes that nucleic acids adsorb onto the lipid nanoparticle surface, forming a distinctive “beads-on-a-string” morphology stabilised by electrostatic interactions between the cationic lipids and anionic nucleic acids.^58,59^ In contrast, the internal encapsulation model suggests complete entrapment of nucleic acids within the lipid core, resulting in multilamellar or vesicular architectures that provide enhanced protection to the genetic payload.^60^ Beyond these primary configurations, specialised lipid formulations like SAINT-2 lipids can generate alternative structural variants such as ellipsoidal genosomes, which form through rapid, thermodynamically favourable self-assembly processes when cationic vesicles complex with pDNA.^61^ These diverse structural paradigms collectively influence the stability, release kinetics and transfection efficacy of genosome-based delivery systems.

## Formation of genosomes

**Figure 2:**
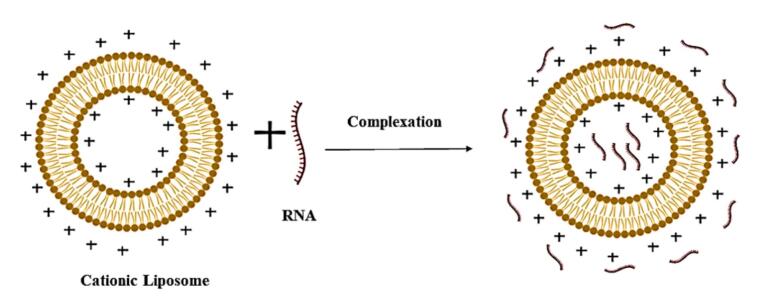
Schematic representation of genosome formation

 Genosomes are synthesised through electrostatic complexation between nucleic acids and cationic lipid formulations, as illustrated in Figure 2.^62^ The assembly process involves mixing lipid and nucleic acid components in an aqueous medium under ambient conditions (20–25 °C), where the positively charged amine groups of cationic lipids interact with the anionic phosphate backbone of nucleic acids, a critical determinant of transfection efficiency.^63^ Empirical studies confirm that higher surface charge density correlates with enhanced transfection capacity due to improved nucleic acid binding affinity.^64^

 The formation mechanism occurs via two distinct thermodynamic phases.^65^ The initial step involves rapid ( < 1 h), exothermic adsorption of nucleic acids onto cationic lipid surfaces, accompanied by counterion release (~90% from lipids and ~70% from nucleic acids), which stabilises the complex through electrostatic bridging.^66,67^ Subsequently, a slower, endothermic reorganisation phase mediates nucleic acid encapsulation within the lipid matrix. This irreversible process entails disruption of hydrophobic lipid domains followed by structural stabilisation via van der Waals forces, ultimately defining the lipoplex architecture and biological performance.^68,69^

 Commonly employed cationic lipids, including DOTMA, DOTAP, DOGS, DODAC, and DODMA, are selected based on their charge density and fusogenicity, while helper lipids (e.g. DOPE, cholesterol (CHOL) enhance membrane stability and cellular uptake.^70,71^ As summarised in Table 5, these lipids exhibit distinct performance profiles. For instance, DOTAP demonstrates moderate transfection efficiency (65±10%) but significant cytotoxicity, whereas ionisable lipids like Dlin-MC3 achieve superior efficiency (80±5%) with reduced toxicity. Particle size, governed by the lipid: nucleic acid charge ratio, critically influences functionality. Near-neutral ratios (slight cationic excess) generate larger complexes with enhanced transfection efficiency, while extreme charge ratios produce smaller but less effective particles.^72^ Notably, excess free liposomes must be removed post-formulation to mitigate cytotoxicity, ensuring optimal safety and therapeutic efficacy.

**Table 5:** Comparative performance of genosome formulations

**Lipid type**	**Particle size**	**Zeta potential**	**Uptake (vs control)**	**Example of formulation**	**Clinical relevance**	**Advantages **	**Limitations **	**Ref. **
Cationic (e.g. DOTAP/DOPE)	~100-150 nm	Highly positive (~ + 30 mV)	3-4x higher	siRNA for lung cancer	Localised therapy (e.g. intratumoral)	Rapid cell entry high DNA loading	Toxic at high doses Unstable in the blood	^73^
Ionisable (e.g. Dlin-MC3)	~80-100 nm	pH-sensitive (neutral in blood, positive in endosomes)	4-5x higher	Onpattro^®^ (hATTR amyloidosis)	Systemic delivery (liver-targeted)	FDA-approved formulations Low immune reaction	Requires cold chain storage Expensive production	^74^
PEGylated Lipids	~150-200 nm	Slightly positive (~ + 10 mV)	2-3x higher	mRNA vaccines (COVID-19 LNPs)	Vaccines/ repeat dosing	Long blood circulation Stealth effect	Adverse effects like PEG allergy in some patients	^75^
Neutral (e.g. DOPE/Chol)	~180-250 nm	Near-neutral	1.5-2x higher	Gene therapy for brain diseases	Neurodegenerative disorders	Very low toxicity Biocompatible	Poor gene release from endosomes	^76^
Targeted (e.g. Folate-PEG)	~90-120 nm	Slightly negative (~ -5mV)	5-6x higher (in cancer cells)	Ovarian cancer therapy	Receptor-positive cancers	Tumour-selective Minimal side effects	Limited to receptor-specific targeting	^77^

## Intracellular delivery mechanisms of genosome-based therapeutics

 The therapeutic efficacy of genosome-mediated gene delivery systems fundamentally depends on their capacity to achieve successful intracellular trafficking and payload release within target cells.^78^ This complex biological process occurs through two mechanistically coupled phases- receptor-mediated cellular internalisation, followed by endosomal escape.^79^ Cellular uptake is initiated when cationic lipoplexes interact with negatively charged cell surface proteoglycans, primarily through clathrin-mediated endocytosis (CME), although alternative pathways, including caveolae-dependent uptake and micropinocytosis, may contribute to varying degrees depending on the specific formulation characteristics. These endocytic mechanisms not only protect the nucleic acid payload from extracellular nucleases but also facilitate transport into the acidic environment of endolysosomal compartments (pH 5.0-6.5), where critical structural transformations occur.^80,81^

**Figure 3:**
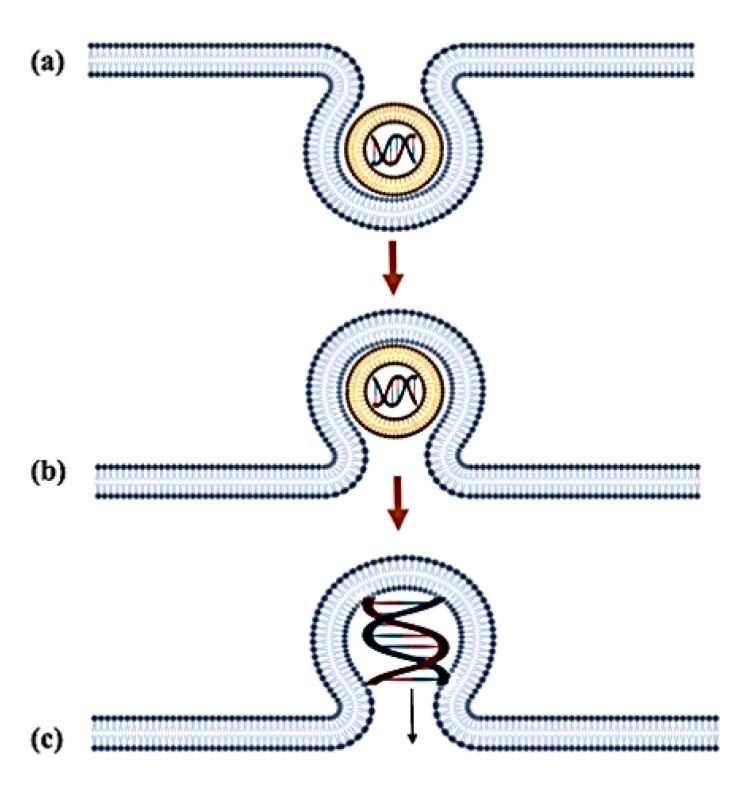
Flip-flop mechanism of the genosome: (a) Fusion of lipoplex with the endosomal membrane (b) Flip-flop of the negatively charged phospholipids (c) Destabilisation of the membrane and release of the nucleic acid

 The endosomal escape process presents the most formidable barrier to successful gene delivery, with two well-characterised mechanisms currently proposed.^82,83^ The flip-flop model involves a sophisticated lipid exchange process where anionic phospholipids (particularly phosphatidylserine) from the inner endosomal membrane leaflet translocate to the outer leaflet through ATP-dependent flippase activity (Figure 3). This translocation creates charge-neutralised ion pairs between the anionic phospholipids and cationic lipids of the genosome, significantly reducing the electrostatic binding affinity for nucleic acids. The resulting charge neutralisation induces a phase separation in the lipoplex structure, ultimately leading to nucleic acid release into the cytosol.^84^ However, this mechanism demonstrates limited efficiency due to kinetic constraints in lipid exchange rates and geometric mismatches between the genosome surface area and available endosomal membrane phospholipids.^85^

**Figure 4:**
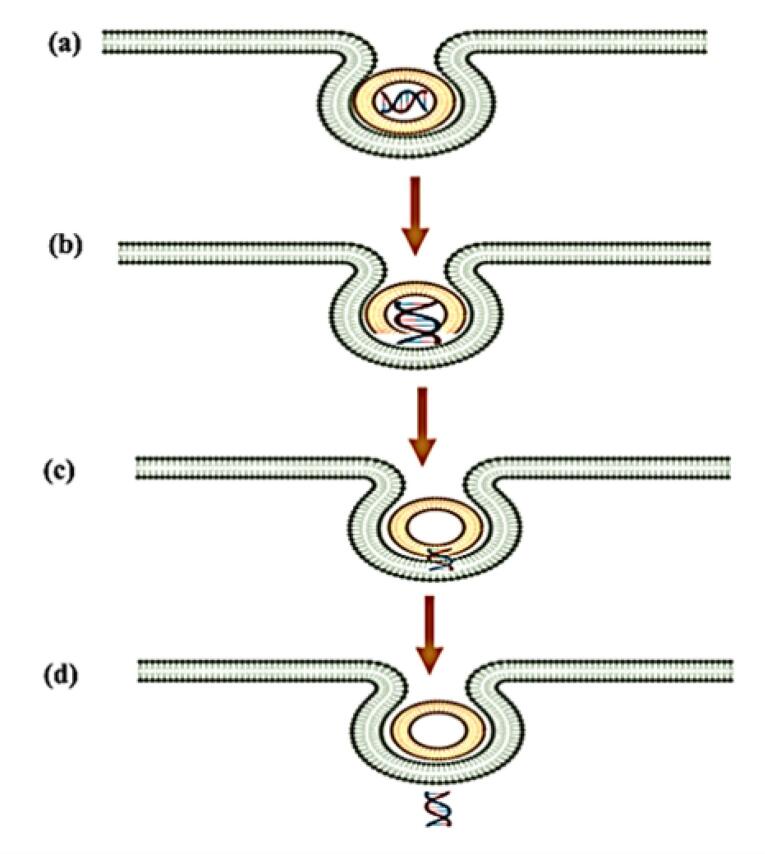
Transient pore formation: (a) Genosome gets localised to the inner-endosomal membrane (b) Dispersal of lipid molecule by lipoplex degradation (c) Endosomal migration of the nucleic acid by transient pore formation (d) Slow release of the nucleic acid after lipid integration

 More recent studies have provided substantial evidence for the transient pore formation mechanism, which involves a multi-step process of membrane destabilisation (Figure 4).^86^ Initially, genosomes localise to the inner endosomal membrane through electrostatic interactions, followed by partial degradation of the lipid-nucleic acid complex in the acidic environment. This degradation induces localised membrane curvature stress, leading to the formation of transient hydrophilic pores (3-10 nm diameters) with lifetimes ranging from milliseconds to seconds. Molecular dynamics simulation suggests these pores are stabilised by the inverted cone-shaped geometry of the helper lipids like DOPE, which reduces the energetic barrier for pore formation. The nucleic acid payload escapes through these transient defects via a combination of electrophoretic forces and concentration gradients, after which the membrane rapidly reseals through lipid rearrangement. This mechanism appears particularly efficient for larger nucleic acid payloads such as pDNA and CRISPR-Cas9 complexes, potentially explaining the superior transfection efficiency observed with certain lipid formulations.

 These sophisticated delivery mechanisms ensure appropriate subcellular localisation of therapeutic nucleic acids while minimising lysosomal degradation. Current optimisation strategies focus on engineering lipid compositions with improved endosomolytic activity, including the development of pH-sensitive ionisable lipids and the incorporation of endosomolytic peptides, while maintaining favourable safety profiles for clinical applications.

###  In vitro/in vivo comparison of endosomal escape mechanisms in genosome delivery

 The flip-flop and transient pore mechanisms represent two distinct but potentially complementary pathways for genosome-mediated endosomal escape, each with unique biophysical characteristics and functional implications. In physiological systems, the flip-flop mechanism dominates, accounting for approximately 60% of release events, as demonstrated by live-imaging studies in primate models.^87^ This process involves the ATP-dependent reorganisation of membrane phospholipids where anionic species translocate across the bilayer to neutralise cationic lipids, ultimately facilitating nucleic acid release. The biological relevance of this mechanism is underscored by its dependence on endogenous lipid recycling pathways that are characteristic of living systems. In contrast, *in vitro *environments favour pore formation, where static culture conditions promote the generation of transient 5-10 nm membrane defects that enable cytosolic entry, as visualised through cryo-EM studies. This pore-mediated escape demonstrates higher efficiency (80% in HeLa cells) but may overestimate delivery potential due to the absence of physiological barriers present in living organisms.^88^

 At the molecular level, these mechanisms exhibit distinct characteristics with important clinical implications. The flip-flop process involves complex lipid rearrangements and salt bridge formation, with FRET-based assays confirming nanoscale reorganisation events. This mechanism’s physiological fidelity makes it particularly valuable for therapeutic development, as evidenced by the superior performance (2-fold higher gene expression) of flip-flop-optimised LNPs in clinical settings.^89^ Meanwhile, pore formation occurs through passive membrane strain, generating short-lived ( < 1 second) leakage pathways detectable by advanced imaging techniques like atomic force microscopy.^90^ Current research reveals these mechanisms may not be mutually exclusive, with emerging hybrid models suggesting pore formation may initiate flip-flop by exposing inner membrane leaflets. Computational studies further indicate potential lipid-protein cooperatively in pore stabilisation, adding complexity to our understanding of these processes.^91^

 Significant questions remain regarding precise molecular requirements and the dynamic interplay of these escape mechanisms in living systems. Key challenges include determining the exact lipid stoichiometry necessary for efficient flip-flop *in vivo *and developing methods to quantitatively track pore dynamics in physiological environments. Recent advances in multiplexed imaging and AI-assisted molecular dynamics simulations are beginning to address these knowledge gaps. These investigations are crucial for optimising next-generation genosome designs, particularly in achieving the delicate balance between efficient endosomal escape and minimal cytotoxicity, a critical factor in translating nanocarrier systems from bench to bedside. The continued elucidation of these escape mechanisms will undoubtedly enhance our ability to engineer more effective and targeted gene delivery systems for diverse therapeutic applications.

###  Functional role of helper lipids in genosome systems 

 Helper lipids serve as critical structural and functional components of genosome formulations, with DOPE and DOPC being most widely utilised for their biomimetic properties and pH-responsive behaviour.^92,93^ DOPE’s ability to undergo lamellar-to-hexagonal (Lα-to-H_II) phase transition under endosomal acidic conditions (pH 5.0-6.5) promotes membrane destabilisation through curvature stress induction and non-bilayer intermediate formation. Molecular interactions between phosphate groups of DOPE and cationic lipid headgroups enhance nucleic acid condensation while facilitating endosomal escape, with optimal activity observed at 30-50 mol% concentrations. Cholesterol further stabilises the lipid bilayer through hydrophobic interactions and modulates membrane fluidity for improved systemic circulation.^94^

 Recent advances have transformed helper lipids from passive structural elements to active functional components through engineered features like pH-triggered conformational switches and intracellular trafficking motifs. These developments enable precise control over genosome stability, biodistribution, and intracellular release kinetics. Current research focuses on synthetic analogues with tunable phase behaviour and reduced immunogenicity, representing a key strategy for optimising the therapeutic index of genosome-based delivery systems while addressing critical translational challenges. The rational design of helper lipid compositions now stands as a fundamental parameter in developing clinically viable nucleic acid therapeutics.

###  Advanced lipid design strategies for enhanced genosome performance 

 Recent innovations in lipid engineering have yielded significant improvements in genosome-mediated transfection efficiency through rational molecular design. A particularly impactful approach involves the development of pH-responsive cationic lipids containing ionisable amine or imidazole groups.^95^ These smart lipids exhibit precisely tuned pKa values (6.2-6.6) that enable charge-state transitions in response to endosomal acidification.^96^ The pH-dependent protonation of these functional groups induces two critical effects- increases electrostatic interactions with endosomal membranes, and structural transitions that promote membrane destabilisation, collectively enhancing nucleic acid release into the cytosol.

 Further optimisation has been achieved through strategic incorporation of unsaturated fatty acid chains (e.g. oleic and linoleic acids) within the lipid architecture.^97^ The cis-double bonds in these hydrocarbon tails introduce kinks that reduce packing density and increase bilayer fluidity. These enhanced membrane dynamics facilitates three key processes- improved fusion with cellular membranes, more efficient disassembly of lipid-nucleic acid complexes, and optimised intracellular trafficking of genetic payloads. Current research focuses on combinatorial approaches that integrate these design principles with targeting ligands and stealth components to develop next-generation genosomes with superior tissue specificity and transfection efficiency.

###  Surface engineering through PEGylation 

 PEGylation, the covalent conjugation of polyethylene glycol (PEG) to genosome surfaces, represents a critical strategy for enhancing systemic circulation and reducing immune clearance.^98^ The hydrophilic PEG chains form a static barrier that minimises opsonisation and recognition by the mononuclear phagocyte system, thereby extending plasma half-life. However, this shielding effect presents a dual challenge- excessive PEGylation can impair critical interactions with cellular membranes, reducing endocytic uptake and intracellular delivery efficiency.^99^

 To address these limitations, advanced PEGylation strategies employ short-chain unsaturated PEG derivatives that balance stealth properties with subsequent detachment in the target microenvironment. These optimised formulations maintain sufficient PEG density for initial immune evasion while allowing timely PEG shedding to facilitate membrane fusion and cellular internalisation. Although this approach may modestly reduce circulation time compared to conventional PEGylation, it significantly enhances transfection efficiency by preserving the genosome’s ability to interact with target cells. Current research focuses on stimuli—responsive PEG lipid conjugates that undergo controlled deshielding in response to tumour microenvironment cues (e.g. pH, enzymes) for improved spatiotemporal delivery control.

###  Physiological barriers in genosome delivery 

 The efficacy of genosome-based gene delivery is governed by their ability to overcome multiple physiological barriers encountered during systemic administration.^100^ Extracellular challenges include enzymatic degradation, serum protein adsorption, and immune surveillance mechanisms that can rapidly clear nanoparticles from circulation. Upon cellular internalisation, genosomes must subsequently navigate a series of intracellular obstacles- plasma membrane penetration, endosomal escape, cytoplasmic trafficking, and, for DNA-based therapeutics, nuclear entry, all while maintaining payload integrity.^101^ A critical additional requirement involves controlled unpacking of the nucleic acid cargo to ensure proper temporal release for optimal gene expression or silencing activity.

 To address these challenges, surface engineering strategies have been developed to improve genosome pharmacokinetics and biodistribution. Charge-shielding modifications using neutral or hydrophilic polymers such as galactose, dextran, or PEG effectively reduce non-specific interactions with plasma proteins, particularly albumin, and minimise immune recognition. These modifications must be carefully balanced to maintain sufficient cellular interaction while preventing rapid clearance. Current optimisation approaches integrate stimuli-responsive elements that maintain stealth properties during circulation but undergo controlled activation at target sites. The successful clinical translation of genosome technology ultimately depends on this multifaceted design paradigm that simultaneously addresses lipid composition optimisation, controlled release mechanism, and physiological barrier evasion, all critical factors for achieving targeted, safe, and effective gene therapy outcomes.

## Parameters influencing physico-chemical properties of genosomes

 The therapeutic performance of genosome systems is fundamentally governed by their transfection efficiency, which is directly modulated by key physicochemical properties including surface charge density, particle size distribution, and colloidal stability.^102^ These characteristics emerge from complex thermodynamic and kinetic interactions during formulation, where critical parameters such as lipid-to-nucleic acid ratio, ionic strength of the medium, mixing kinetics, and complexation temperature must be precisely controlled. The lipid-to-DNA charge ratio represents a particularly crucial thermodynamic parameter that dictates the structural organisation of resulting lipoplexes- highly cationic complexes achieve complete nucleic acid condensation, while anionic formulations often contain unbound DNA strands.^103^ Neutral charge ratios (zeta potential ~ 0) typically produce heterogeneous size distributions due to diminished interparticle electrostatic repulsion, leading to aggregation and compromised stability.

 Advanced empirical approaches have been developed to control genosome characteristics through careful manipulation of preparation conditions.^104^ Charge-directed assembly methods demonstrate that adding nucleic acids to preformed lipid dispersions yields positively charged complexes, while inverse addition produces anionic systems. Mixing kinetics significantly influence particle morphology, with rapid mixing generating smaller, more uniform lipoplexes compared to the aggregated structures formed during slow mixing. While temperature effects are generally minimal for DNA stability, they may modulate complexation kinetics. Ionic strength represents another critical variable, where elevated salt concentrations can both promote component association through charge screening while potentially destabilising colloids. These formulation parameters must be systematically optimised to achieve the delicate balance between nucleic acid protection, cellular uptake efficiency, and intracellular release kinetics required for effective lipofection. Current research focuses on computational modelling approaches to predict optimal formulation conditions based on molecular interaction parameters, representing a promising direction for rational genosome design.

## Application of genosomes

 Genosome-based therapeutics have achieved notable clinical success since their first regulatory approval in 2003, when China approved Gendicine for the treatment of head and neck squamous cell carcinoma.^105,106^ These lipid-based non-viral vectors represent a paradigm shift in gene therapy, particularly for genetic disorders and refractory cancers where conventional treatments prove inadequate. Their design leverages cationic lipids to condense nucleic acids while mimicking viral delivery mechanisms, offering distinct advantages including reduced immunogenicity, lower toxicity profiles, and improved targeting capabilities compared to viral vectors- albeit with generally lower transfection efficiency.^107^ The clinical development of genosomes has progressed significantly across multiple therapeutic areas through innovative formulation strategies.

 In pulmonary medicine, genosomes enable cell-specific delivery through optimised administration routes. Intranasal delivery of SPC-targeted miRNA lipoplexes achieves selective transfection of alveolar type II pneumocytes, demonstrating enhanced local action at the airway epithelium.^108^ The multifunctional envelope-type nano-device (MEND) represents a significant advancement, with the optimised YS05-MEND formulation showing superior efficacy against lung metastases compared to conventional chemotherapy in preclinical models.^109,110^ These systems combine lipid, polymer, and protein components to enhance cytosolic delivery of RNA therapeutics while maintaining favourable safety profiles.

 Genosome technology shows particular promise for cutaneous applications where localised delivery is paramount. For radiation-induced skin damage, PUMA siRNA incorporated into Carbopol hydrogels enables epidermal-specific RNAi delivery while sparing deeper tissue layers.^111^ Co-formulation with DOTAP further enhances transdermal delivery to epidermal melanocytes, demonstrating the importance of lipid composition in tissue penetration.^112^ Hair follicle gene therapy represents another innovative application, with *in vivo *murine studies achieving up to 50% transfection efficiency in progenitor cells following depilation and retinoic acid pretreatment.^113^ These applications highlight the critical relationship between formulation parameters such as lipid-to-DNA ratio and absolute concentrations and therapeutic outcomes.^114^

 Genosomes address the formidable challenge of blood-brain barrier (BBB) penetration for neurological applications. Lipoplex systems combining lipid and polymer components enable non-invasive delivery of therapeutic genes to the CNS while maintaining high loading capacity and production scalability.^115,116^ Advanced targeting strategies include transferrin receptor-targeted immunoliposomes, which in recent studies restored striatal tyrosine hydroxylase activity in Parkinson’s disease models.^117,118^ For Alzheimer’s disease (AD), chitosan-precondensed lipoplexes delivering ApoE2 plasmids show therapeutic potential, with bifunctionalised systems (mApoEPA-LIP) demonstrating reduced amyloid burden and cognitive improvement in transgenic models.^119,120^ Surface modification targeting GLUT1 transporters and incorporating rabies virus glycoprotein derivative further enhances CNS penetration.^121^

 Genosome formulations overcome significant pharmacological challenges in treating parasitic infections. Dinitroaniline compounds, while effective against Leishmania species, benefit from PC-based encapsulation to address poor solubility and stability issues.^122^ In malaria therapeutics, soy-PC and cholesterol lipoplexes containing monensin demonstrate enhanced activity against both *Plasmodium berghei* in murine models and *Plasmodium falciparum in vitro*.^123,124^ These applications underscore the importance of lipid composition of genosomes in improving drug bioavailability and therapeutic index for infectious diseases

 RNA-lipoplexes represent a breakthrough in cancer immunotherapy, with several candidates reaching clinical trials. These formulations specifically target splenic dendritic cells to enhance antigen presentation, as demonstrated in B16 melanoma models where tumour progression was significantly suppressed.^125,126^ The structural transition from RNA-cationic liposome complexes to RNA-lipoplexes provides critical protection against nucleases while improving cellular uptake. Vascular-targeted siRNA lipoplexes show similarly promising results, addressing the rapid clearance limitations of free siRNA through enhanced tissue retention and favourable pharmacokinetics without observed toxicity.

## Advances in genosome therapeutics: Clinical progress, delivery challenges and patent landscape

 Genosome-based therapies have revolutionised precision medicine. With landmark approvals such as Patisiran (Onpattro^®^) for hereditary transerythrin amyloidosis (hATTR) and mRNA-LNP vaccines for COVID-19, these platforms now target oncology, genetic disorders, and infectious diseases. However, challenges in delivery efficiency, immunogenicity, and manufacturing scalability persist.

###  Clinical progress in genosome therapeutics

 Recent years have witnessed transformative clinical advancements in genosome-based therapeutics, particularly in oncology, genetic disorders, and infectious diseases. In oncology, siRNA and mRNA platforms have demonstrated promising antitumour activity, albeit with challenges in tolerability. For instance, a Phase I trial investigating LNP-encapsulated KRAS-targeting siRNA in pancreatic cancer reported significant tumour regression in 30% of patients, although dose-limiting cytokine release syndromes necessitated careful dose optimisation.^127^ Similarly, personalised neoantigen mRNA vaccines have shown enhanced T-cell responses in melanoma, underscoring the potential of mRNA-LNPs in cancer immunotherapy. Beyond oncology, CRISPR-based therapies for hereditary transthyretin amyloidosis achieved substantial serum TTR reduction, yet liver toxicity remains a critical barrier to broader application.^128^

 The clinical development of genosome therapies remains highly skewed toward early-phase trials, with over 80% of ongoing studies in Phase I/II. This trend reflects the emphasis on safety assessments, particularly for novel modalities like CRISPR and tumour-targeted siRNA. However, notable late-stage successes, such as Patisiran’s approval for hATTR and the rapid deployment of mRNA-LNP COVID-19 vaccines, validate the translational potential of these platforms.^129^ The APOLLO trial established Patisiran’s 0.3 mg/kg every-three-weeks regimen as optimal, achieving an 81% reduction in pathogenic TTR levels, while the COVID-19 vaccines highlighted the pivotal role of ionisable lipids (e.g. SM-102, ALC-0315) in enhancing efficacy and stability.^130^

 Despite these successes, key challenges persist, including cytokine-driven toxicities in oncology applications, immunogenicity of PEGylated lipids, and limited extrahepatic delivery efficiency.^131^ Table 6 summarises recent landmark clinical trials, illustrating the therapeutic scope and unresolved hurdles in genosome development. Addressing these limitations through innovative delivery strategies and robust safety monitoring will be critical for advancing next-generation candidates into late-phase trials and clinical practice.

**Table 6:** Current status of genosome-based therapies in clinical trials

**Delivery system**	**Gene**	**Delivery route**	**Indications**	**Development phase**	**Status**	**Sponsor**	**ClinicalTrials.gov identifier**	**Ref. **
Pbi-shRNA lipoplex	EWS/FLI1 gene (driver gene of Ewing’s sarcoma)	Intravenous	Advanced Ewing’s sarcoma	1	Active, not recruiting	Gradalis, Inc.	NCT02736565 (2023)	^132^
Pbi-shRNA lipoplex	STMN 1 gene (leukaemia-associated cytoplasmatic phosphoprotein)	Intratumoral	Advanced and/or Metastatic cancer	1	Completed	Gradalis, Inc.	NCT01505153 (2018)	^133^
Tetravalent RNA-lipoplex	NY-ESO-1 (New York ESO-1) MAGE-A3 (Melanoma associated antigen A3) TPTE (trans-membrane phosphatase with tensin homology) Tyrosinase	Intravenous	Advanced Melanoma	1	Active, not recruiting	BioNTech SE	NCT02410733 (2023)	^134^
Immune-tethered lipoplex nanoparticle (ILN) biochip			Diffuse large cell B-lymphoma	NA	Recruiting	Ohio State University Comprehensive Cancer Centre	NCT03656835 (2022)	^135^

###  Delivery challenges and solutions in genosome therapeutics

 The clinical translation of genosome-based therapeutics faces significant hurdles related to delivery routes, each presenting unique limitations. Intravenous (i.v.) administration, while widely used, is hampered by the accelerated blood clearance (ABC) phenomenon, where repeated dosing triggers immune-mediated clearance of LNPs, reducing therapeutic efficacy.^136^ Additionally, passive liver sequestration, driven by endogenous opsonisation, limits extrahepatic targeting, necessitating strategies such as surface charge modulation or pre-dosing with empty LNPs to mitigate undesired biodistribution. Localised delivery, particularly in solid tumours like pancreatic cancer, encounters anatomical barriers such as dense stromal tissue and hypovascularisation, which impede nanoparticle penetration. Emerging solutions, including convection-enhanced delivery and stromal disruption agents, are being explored to enhance tumour accessibility. Inhaled genosomes, though promising for respiratory diseases, face rapid clearance by the mucociliary mechanism and alveolar macrophages, prompting innovations in mucoadhesive coating and sustained-release formulations to prolong lung residency.

 Beyond route-specific challenges, technical hurdles in formulation design and manufacturing further complicate genosome development. PEGylation, a common strategy to prolong circulation, paradoxically induces anti-PEG antibodies in up to 40% of patients, leading to hypersensitivity reactions and reduced efficacy upon repeat dosing.^137,138^ Alternative stealth coatings, such as polyzwitterions and polysarcosine, are under investigation to circumvent immunogenicity while maintaining pharmacokinetic benefits. Scalability remains another critical bottleneck, as batch-to-batch variability in LNP size, encapsulation efficiency, and stability can compromise clinical outcomes. Quality-by-Design (QbD) approaches, coupled with microfluidic manufacturing, are being adopted to enhance reproducibility, yet regulatory alignment on critical quality attributes is still evolving.

 Active targeting strategies, though promising, require precise optimisation of ligand density to balance binding avidity and systemic clearance. For instance, folate and RGD peptide-decorated genosomes have demonstrated enhanced tumour accumulation in preclinical models, but excessive ligand loading can trigger off-target uptake or aggregation.^139^ Recent advances in computational modelling and high-throughput screening are refining ligand-conjugation protocols to achieve optimal targeting efficiency. Furthermore, the integration of stimuli-responsive linkers aims to improve site-specific payload release. Collectively, addressing these delivery challenges and regulatory science will be pivotal for realising the full therapeutic potential of genosomes in diverse clinical settings.

###  Translational roadmap for genosome therapeutics

 The successful clinical translation of genosome-based therapies requires robust monitoring technologies to evaluate biodistribution and therapeutic efficacy. Recent advances in molecular imaging have enabled real-time tracking of genosomes using positron emission tomography (PET) with ^64^Cu-labelled nanoparticles, providing critical insights into their pharmacokinetics and tissue accumulation patterns.^140,141^ Additionally, cell-free RNA (cfRNA) in liquid biopsies has emerged as a promising biomarker for assessing delivery efficiency and target engagement, offering a non-invasive approach to monitor treatment response. These innovations in therapeutic monitoring are complemented by developments in bioanalytical methods, including advanced spectroscopy and chromatography techniques, which enhance the characterisation of genosome formulations and their biological interactions. Together, these tools are paving the way for more precise and personalised therapeutic regimens.^142^

 From a regulatory perspective, the approval pathway for genosome therapies is shaped by evolving guidelines from the FDA and EMA, which specify CQAs such as particle size, polydispersity index, and encapsulation efficiency. Notably, regulatory classifications differ significantly between CRISPR-based gene editing and siRNA/mRNA therapies, with the former facing more stringent scrutiny due to permanent genomic modifications. Furthermore, safety assessments require comprehensive chemistry, manufacturing, and controls (CMC) data, particularly for lipid excipients, to address potential immunogenicity and toxicity concerns. Harmonising these regulatory standards across jurisdictions remains a challenge, necessitating ongoing dialogue between developers and agencies to streamline the approval process while ensuring patient safety.^143^

 The patent landscape for genosome technologies reflects rapid innovation in delivery systems and therapeutic applications. Recent filings highlight advancements in ionizable lipid designs, targeted ligands, and scalable manufacturing processes, with a growing emphasis on modular platforms that can be adapted for multiple disease indications. However, intellectual property disputes and overlapping claims pose potential barriers to commercialisation, underscoring the need for clear strategies early in development. Table 7 summarises key recent patents in the field, illustrating the diversity of technological approaches and their assignees. As the field matures, collaboration between academia, industry, and regulators will be essential to translate these innovations into clinically viable therapies that address unmet medical needs.

**Table 7:** Recent patent works on lipoplexes

**Patent No.**	**Filing country**	**Title of work**	**Granted year**	**Ref**
US10705085B2	United States	Tethered lipoplex nanoparticle biochips and methods of use	2020	^144^
RU2671857C1	Russia	New method for production of lipoplex for local introduction and anti-tumor medication that uses such lipoplex	2018	^145^
RU2784928C2	Russia	Preparation and storage of liposomal RNA compositions suitable for therapy	2022	^146^
EP3427723B1	European Patent Office	RNA formulation for immunotherapy	2020	^147^
JP6980230B2	Japan	New branched chain amphipathic lipids	2021	^148^
JP2018531239A6	Japan	Novel branched-chain amphiphilic lipids	2021	^149^
EP2998289B1	European Patent Office	Compounds for targeting drug delivery and enhancing siRNA activity	2019	^150^
JP6383480B2	Japan	Amine-containing transfection reagents and methods for producing and using the same	2018	^151^
US11124582B2	United States	FLT3L-FC fusion proteins	2021	^152^
US10662060B2	United States	Manufacture of lipid-based nanoparticles using a dual asymmetric centrifuge	2020	^153^
JP6905469B2	Japan	Superbranched polymers and polyplexes, and DNA or RNA delivery systems containing them	2021	^154^
KR102264820B1	South Korea	Stable formulations of lipids and liposomes	2021	^155^

## Future perspectives

 Genosome technology has emerged as a transformative platform for nucleic acid delivery, offering distinct advantages in biocompatibility, scalability, and structural versatility compared to viral vectors. However, several critical challenges must be addressed to bridge the gap between preclinical promise and clinical translation. A fundamental limitation remains the incomplete characterisation of genosome-biological system interactions, particularly regarding cellular internalisation mechanisms, intracellular trafficking patterns, and endosomal escape efficiency. Advanced molecular imaging modalities, including single-particle tracking microscopy and super-resolution imaging techniques, coupled with high-throughput screening platforms, could provide unprecedented insights into these processes at nanometer resolution. Such fundamental understanding will enable the rational design of next-generation formulations with enhanced delivery efficiency.

 The expanding therapeutic potential of RNA-based medicines presents both opportunities and formulation challenges. While genosomes have demonstrated success with single oligonucleotide delivery, the co-encapsulation of multiple RNA species (e.g. siRNA-mRNA combinations) remains technically demanding. Emerging microfluidic production platforms and AI-driven formulation algorithms show particular promise for optimising these complex delivery systems. From a translational perspective, critical pharmaceutical challenges, including long-term stability, lyophilisation compatibility, and prevention of particle aggregation, require systematic investigation. The phenomenon of ABC observed with PEGylated formulations necessitates exploration of alternative stealth technologies, such as zwitterionic polymers or polyglycerol coatings, to enable repeated administration regimens.

 Structural optimisation represents a crucial development pathway, with particular emphasis on chemically-defined, biodegradable lipid systems. The design of ionisable lipids with precisely tuned pKa values could significantly improve pH-responsive behaviour and endosomal escape kinetics. Complementary advances in helper lipid chemistry, including the development of zwitterionic phospholipid analogues, may enhance both formulation stability and intracellular delivery efficiency. Targeted delivery strategies continue to evolve, with multifunctional ligand systems (e.g. transferrin-folate conjugates) showing enhanced specificity for challenging therapeutic targets like the BBB. The integration of stimulus-responsive elements (pH, enzyme or redox-sensitive linkers) could further enable disease site-specific activation of therapeutic payloads.

 From a manufacturing perspective, the transition to GMP-compliant production using continuous-flow microfluidic systems will be essential for clinical-scale implementation. This must be accompanied by the development of robust analytical characterisation methods to ensure batch-to-batch reproducibility. Comprehensive safety assessment protocols need to address potential immunogenicity concerns and establish detailed biodistribution profiles across relevant disease models. The field would benefit from harmonised regulatory guidelines specific to lipid-based gene delivery systems, covering aspects from physicochemical characterisation to clinical evaluation criteria.

 In conclusion, while significant challenges remain, the continued evolution of genosome technology holds tremendous promise for addressing unmet needs across diverse therapeutic areas, including oncology, neurodegenerative disorders, and genetic diseases. By systematically addressing current limitations in formulation science, delivery efficiency, and manufacturing scalability, genosome-based therapies may soon achieve their potential as clinically transformative modalities. The coming decade will likely witness exciting advances as these sophisticated delivery systems transition from laboratory innovation to clinical reality, potentially revolutionising the field of gene therapy.

## Ethical and translational considerations

 As genosome therapies advance toward clinical application, several ethical considerations merit careful deliberation. These include equitable access to advanced therapies, long-term monitoring of potential off-target effects, and appropriate patient selection criteria. The scientific community must proactively address these concerns through transparent research practices and collaborative engagement with regulatory bodies. Establishing international consensus on manufacturing standards and quality control parameters will be crucial for ensuring both patient safety and therapeutic efficacy. Furthermore, interdisciplinary collaboration spanning lipid chemistry, molecular biology, pharmaceutical sciences, and clinical medicine will be essential to fully realise the potential of genosome technology.

## Conclusion

 Genosomes have established themselves as a structurally refined category of non-viral delivery systems, demonstrating remarkable adaptability for nucleic acid therapeutics. These lipid-based nanocarriers, incorporating cationic lipids, helper phospholipids, and surface-modified components, provide effective genetic material protection while enabling crucial biological interactions including cellular uptake, endosomal membrane disruption, and intracellular transport. Their superior safety characteristics, notably diminished immunogenic responses and cellular toxicity relative to viral vectors, make them particularly suitable for treating various pathological conditions spanning oncological, neurological, and genetic disorders. While considerable progress has been achieved, several translational hurdles remain to be overcome. Persistent challenges include optimising gene transfer efficiency, enhancing tissue-specific delivery precision, and resolving formulation stability concerns. Future research directions should emphasise intelligent lipid design with environment-responsive properties, development of composite delivery platforms, and refinement of nucleic acid encapsulation methodologies. Incorporation of molecular targeting ligands combined with advanced screening technologies will facilitate the creation of customised therapeutic solutions. For successful clinical translation, three pivotal elements require attention: implementation of scalable production protocols, comprehensive safety evaluation, and establishment of appropriate regulatory guidelines. Emerging data indicate that genosomes possess the potential to become a transformative gene therapy platform, combining favourable safety profiles with the ability to overcome critical delivery obstacles. Continued innovation in this field may provide solutions for numerous unmet medical needs across multiple therapeutic areas, representing a significant advancement in nucleic-acid-based medicine.

## Competing Interests

 The authors report no financial or any other conflicts of interest in this work.

## Ethical Approval

 Not applicable.
